# Computational modeling of the rectangular non-aligned multi-injector for efficient fuel mixing in a supersonic combustion chamber

**DOI:** 10.1038/s41598-024-66309-1

**Published:** 2024-07-10

**Authors:** Pan Zhang, Zhen Li, Seyyed Amirreza Abdollahi

**Affiliations:** 1 School of Mechanical Engineering, Sichuan University Jinjiang College, Pengshan, 620860 Sichuan China; 2 Department of Automotive Engineering, Sichuan Vocational and Technical College of Communications, Chengdu, 611130 Sichuan China; 3 Faculty of Mechanical Engineering, University of Tabriz, Tabriz, Iran

**Keywords:** Supersonic combustion, Injection system, Cross-jet, CFD, Aerospace engineering, Mechanical engineering

## Abstract

The present investigation examines the usage of rectangular multi-injectors for fuel injection in a supersonic combustion chamber. To evaluate the fuel jet penetration and distribution, a computational method is applied to model the supersonic compressible flow with cross multi-fuel jets released from annular rectangular nozzles with different nozzle configurations. The main effort of this work is to evaluate the jet interactions in the existence of cross-supersonic flow. Fuel jet penetration and distribution are evaluated for three proposed injector arrangements to attain the more efficient option for better fuel mixing. Our results show that reducing injector space improves fuel mixing inside the combustor via creation of strong vortices. Beside, injection of air from internal nozzle increase fuel interactions and fuel mixing inside combustion chamber.

## Introduction

In the realm of advanced aerospace propulsion systems, particularly in the development of scramjet engines, optimizing the fuel injection process is critical for achieving efficient combustion and high performance. The design of fuel injectors, including their shape and arrangement, plays a pivotal role in determining fuel penetration and mixing within the combustor of a scramjet engine. These factors directly influence combustion efficiency, flame stability, and ultimately, the overall performance of the engine^1,2^.

The shape and arrangement of fuel multi-jet injectors play a crucial role in optimizing fuel penetration and mixing within the combustor of a scramjet engine. Scramjet engines are an advanced type of air-breathing propulsion system used in hypersonic flight, capable of achieving speeds greater than Mach 5. Achieving efficient and effective fuel injection is essential for the performance and overall combustion process of scramjet engines^3,4^.

Fuel penetration refers to the distance traveled by fuel droplets injected into the combustor before they vaporize and mix with the incoming air. Effective fuel penetration ensures that the fuel is adequately mixed with the surrounding air, promoting efficient combustion and maximizing thrust production. On the other hand, poor fuel penetration can lead to incomplete combustion, reduced performance, and increased emissions^5,6^.

The arrangement and shape of fuel multi-jet injectors significantly impact fuel penetration and mixing. The arrangement refers to the spatial distribution and positioning of the injectors within the combustor, while the shape refers to the geometry of the injector nozzles and the spray pattern they produce. By optimizing these factors, engineers can enhance fuel penetration, promote fuel–air mixing, and ultimately improve the overall performance of the scramjet engine^7–9^.

To evaluate and improve the fuel injection techniques within scramjet engines, computational fluid dynamics (CFD) has emerged as a valuable tool. CFD enables detailed numerical simulations of fluid flow and combustion processes, allowing engineers to analyze and optimize the performance of new innovative injection techniques. By using CFD, engineers can study the behavior of fuel sprays, analyze the interaction between fuel droplets and the surrounding air, and assess the resulting fuel–air mixing and combustion characteristics^10,11^.

The usage of CFD for evaluating new innovative injection techniques provides several advantages. It allows for a cost-effective and time-efficient evaluation of different injector designs, reducing the need for extensive experimental testing. CFD simulations can provide valuable insights into the complex flow physics occurring within the combustor, enabling engineers to make informed decisions regarding the fuel injection system^12–14^.

The shape and arrangement of fuel multi-jet injectors have a significant impact on fuel penetration and mixing within the combustor of a scramjet engine. Optimizing these factors is crucial for achieving efficient combustion and maximizing the performance of the engine. CFD simulations have become indispensable for evaluating new innovative injection techniques, providing valuable insights into fuel–air mixing and combustion characteristics. By leveraging CFD, engineers can continually strive to improve fuel injection systems and enhance the overall efficiency of scramjet engines^15–17^.

Fuel injectors in scramjet engines are tasked with delivering fuel into the high-speed airflow of the combustor, where conditions are characterized by extreme velocities and temperatures. The design and configuration of these injectors must be carefully tailored to ensure effective fuel atomization, rapid mixing with the air, and ignition under such demanding conditions^18,19^.

The shape and arrangement of fuel multi-jet injectors are key parameters that significantly impact fuel spray characteristics. The choice of injector shape influences factors such as spray angle, droplet size distribution, and spray momentum, which collectively affect how well the fuel mixes with the incoming air. Additionally, the spatial arrangement of multiple injectors within the combustor determines the distribution and interaction of fuel sprays, which in turn affects the overall combustion process^20,21^.

To study and optimize the performance of new innovative injection techniques for scramjet engines, Computational Fluid Dynamics (CFD) has emerged as a powerful tool. CFD allows engineers and researchers to simulate the complex flow phenomena and combustion processes occurring within the combustor, taking into account the intricate interactions between fuel injection, mixing, and combustion^22,23^.

By leveraging CFD simulations, engineers can evaluate and refine different injector designs and arrangements virtually, before physical prototypes are constructed. This enables rapid iteration and optimization of fuel injection strategies without the need for costly experimental setups. CFD analysis provides detailed insights into fuel penetration depths, spray dispersion, and mixing efficiency under varying operating conditions, thereby guiding the development of more effective injector configurations^24–26^.

In this context, the integration of advanced CFD techniques with innovative fuel injector designs represents a promising approach towards enhancing the performance and efficiency of scramjet engines. This paper explores the effects of different injector shapes and arrangements on fuel penetration and mixing within the combustor, highlighting the importance of CFD as a valuable tool for the evaluation and optimization of these critical components in next-generation aerospace propulsion systems. The subsequent sections will delve into specific aspects of injector design and CFD methodology, elucidating their roles in advancing the state-of-the-art in scramjet technology.

**Figure 1:**
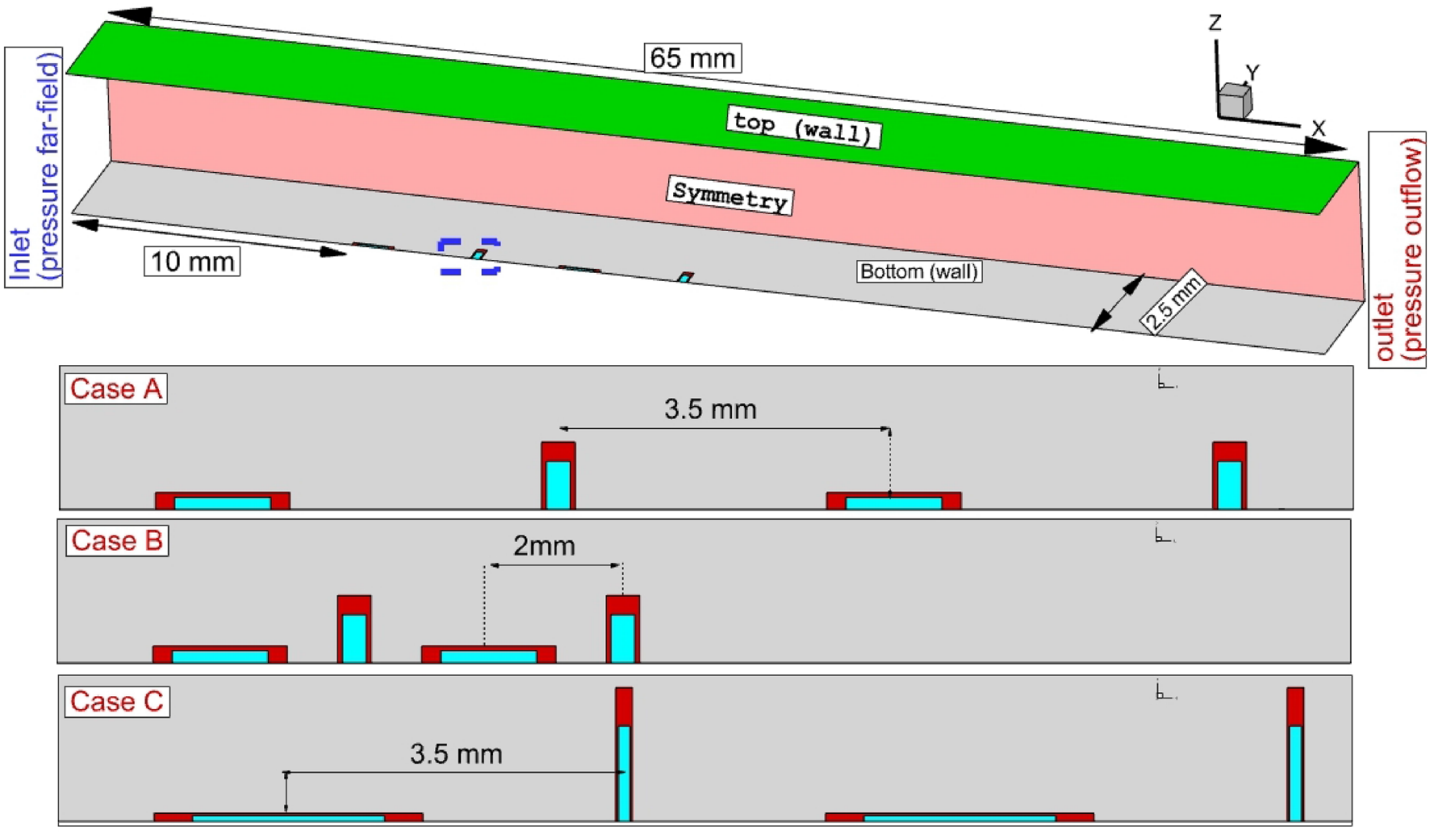
Injector shape and arrangements.

As demonstrated in Fig. 1, this study has analyzed three rectangular nozzle configurations at the supersonic cross stream with Mach = 4. The fuel jet is released in front of the annular nozzle while air jets are also employed to improve the fuel jet interactions. The computational technique of CFD is developed for the modeling of the fuel jet released from suggested nozzle arrangements. The interactions of the fuel jet with free stream cross-flow are examined via Mach and mass contour of the fuel jets. The size of the fuel mixing zone and the penetration height are also compared in these jet systems to disclose the role of the induced circulation on the distribution of the multi-fuel jet arrangements.

## Main governing equations and computational methodology

RANS equations are the main governing equations for computational modeling of the compressible flow inside the combustion chamber^27–29^. Since the attention of this work is to investigate the role of the modified injector arrangement on the fuel distribution, reactions are not considered and the analysis of the fuel diffusion is done. The flow gas is assumed ideal and the SST turbulence model is applied for the estimation of the viscosity in our study^30–32^.

The SST (Shear Stress Transport) turbulence model is a widely used two-equation model for simulating turbulent flows. It was developed by F.R. Menter to effectively blend the robust and accurate formulation of the k-omega model in the near-wall region with the free-stream independence of the k-epsilon model in the outer region of the boundary layer. This model is particularly well-suited for a wide range of turbulent flow simulations, including those with adverse pressure gradients, separation, and reattachment. When it comes to simulating supersonic flows in a combustion chamber, the SST model can be a good choice, especially if the flow involves complex interactions between turbulence, chemistry, and heat release^33–35^.

Hydrogen gas is chosen as fuel and consequently, species mass transport is also coupled with the main governing equations. The energy equation is also required for the modeling of compressible flow since the shock waves are produced in the selected domain^36–38^.

Figure 1 displays the schematic of the selected model with applied conditions on the boundary. The inlet supersonic airflow enters to domain at Mach = 4 with a static temperature of 1000 K and 1 atm pressure. The hydrogen fuel jet is released with 10% total pressure of the free stream supersonic air stream. The velocity of the injected hydrogen jets is sonic. As illustrated in Fig. 1, the top and bottom of the model are assumed walls, and two sides are considered symmetrical. Pressure far-field and outlet pressure is applied for the inlet and outlet, respectively. The size of the fuel jet nozzle is also displayed in Fig. 1. The jet space for Case A and Case C is equal to 3.5 mm to evaluate the importance of the nozzle aspect ratio on the fuel mixing of the proposed injection system. The surface area of the outer annular nozzle is equal to the inner jet. The surface area of both the internal and annular rectangular nozzle is equal to the circular nozzle with a diameter of 0.5 mm. In case B, the gap of the nozzle is reduced to 2 mm to compare the significance of the jet space on the flow and mixing patterns. The total length of the domain is 65 mm and the fist injector is positioned 10 mm behind the inlet. To reduce the computational cost, half of the domain is simulated^39–41^.

**Figure 2:**
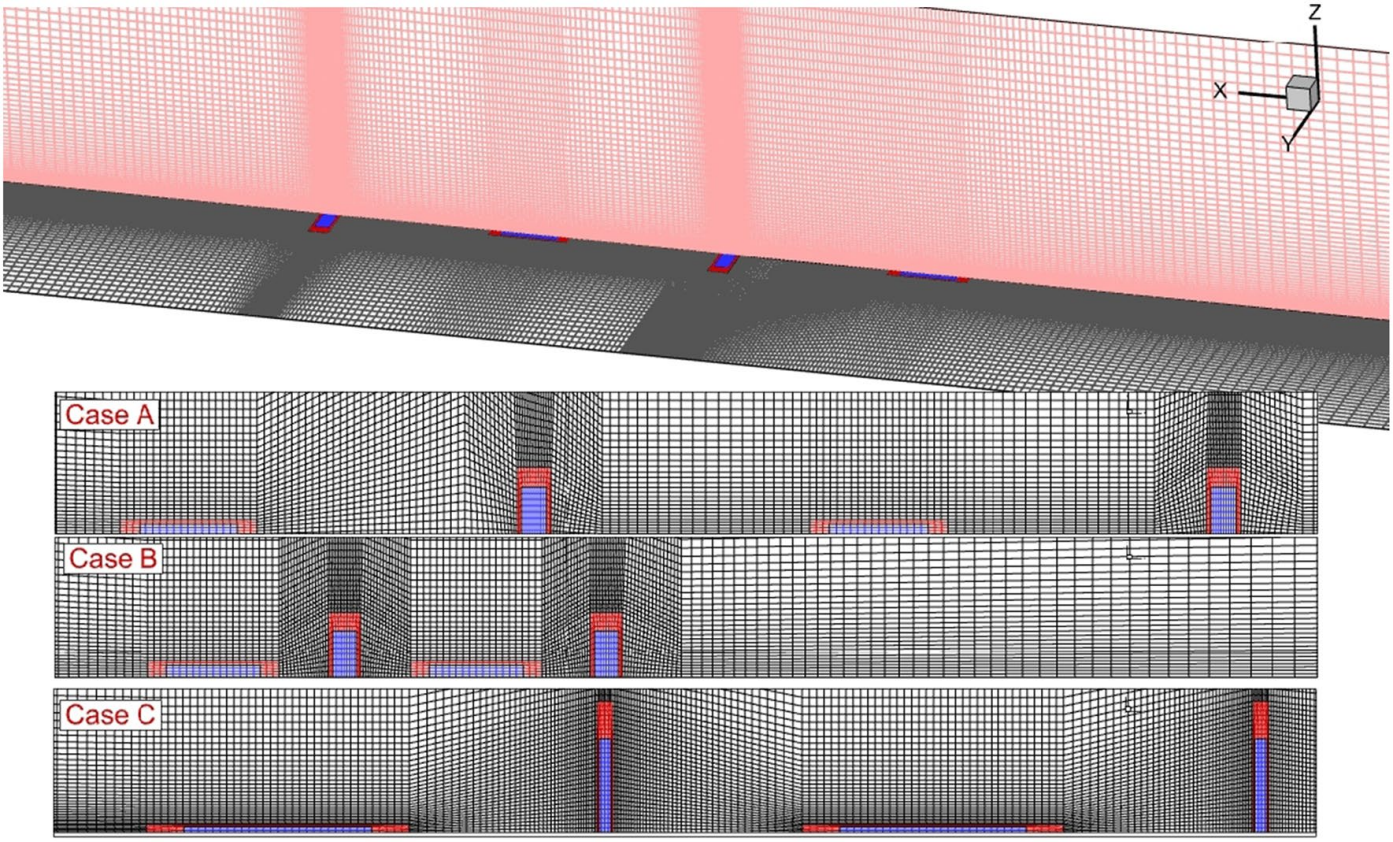
Generation of grids.

The generated grid for each proposed jet configuration is displayed in Fig. 2. The structured grids are produced in which calculated results is more reliable. Besides, the grid size is also changed inside the domain based on the importance of the flow and the probable location of shock interactions. The grid study is also conducted to evaluate the independence of attained results from produced grids. Thus, four grids are produced for this purpose and the mean concentration of the hydrogen on the specific planes positioned I5 mm behind the first jet of case A is compared the grid is sufficiently refined near the walls of the injector to Ensure accurately capture the boundary layer. This typically involves using a fine mesh near the walls to resolve the viscous sublayer and the transition to the turbulent region. The region near the injector where fuel and oxidizer are introduced into the combustion chamber should be highly resolved to capture the initial mixing and breakup of the fuel jets. The performed evaluation indicates that the model with 968,000 cells is a reasonable selection for our research work.

## Results and discussion

The comparison of the numerical data of the present study and the available experimental data is also required for the validation of the employed computational method^42^. As presented in Table 1, the value of the penetration height is compared with other reported data on the symmetry plane. The comparison of the results is related to the model with a single injector with a circular shape at Mach = 4. The change in the achieved results also authenticates the accuracy of the applied numerical approach for the modeling of the compressible supersonic stream with transverse jet flow.

**Table 1:** Comparison of penetration height.

Distance behind the jet (mm)	Computational data of Pudsey et al. (mm)^42^	Our data (mm)
10	8.1	7.78
20	8.45	8.32
30	8.63	8.58
40	9.58	9.45
50	9.88	9.66

**Figure 3:**
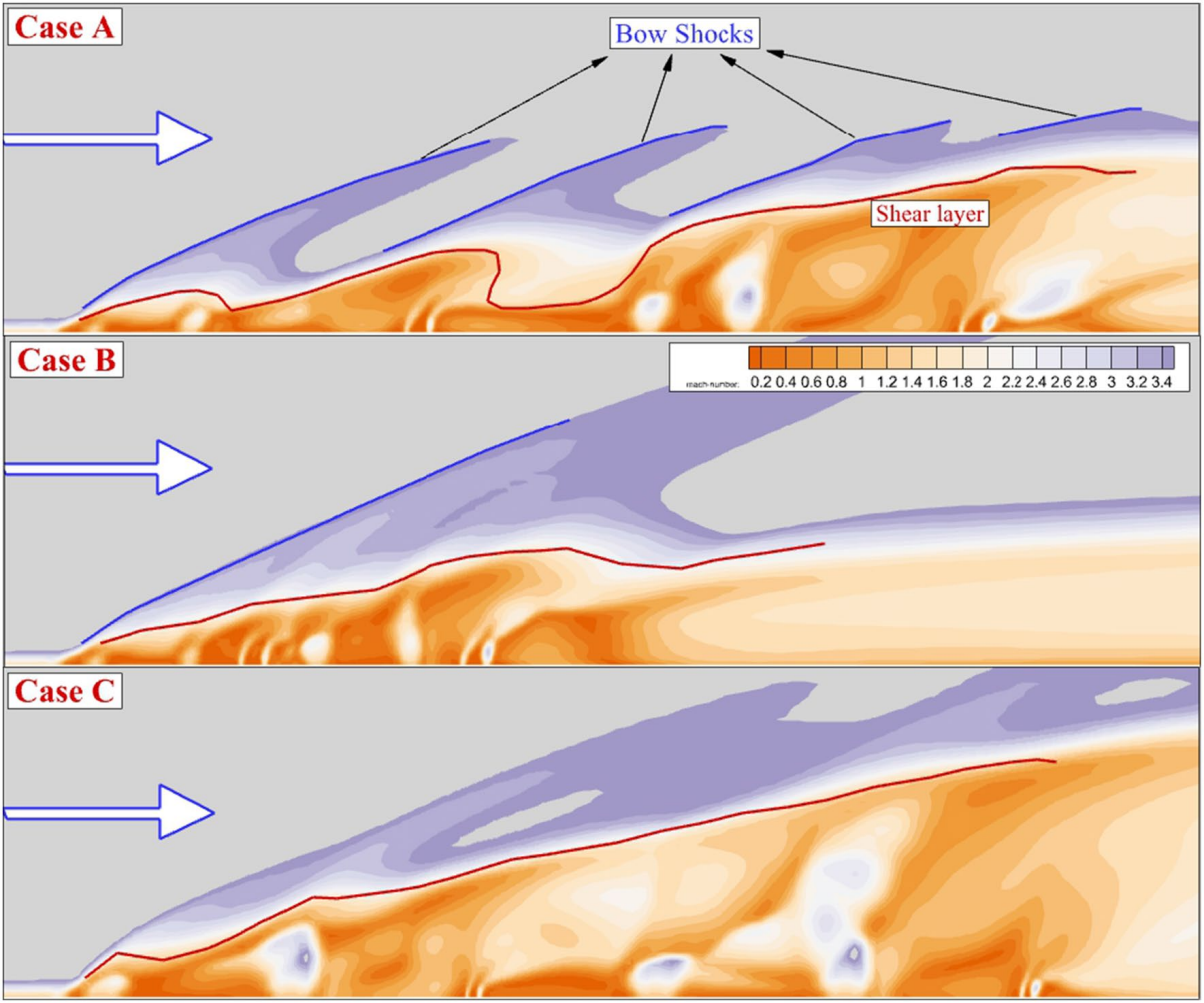
Mach contour on jet plane of annular configuration.

**Figure 4:**
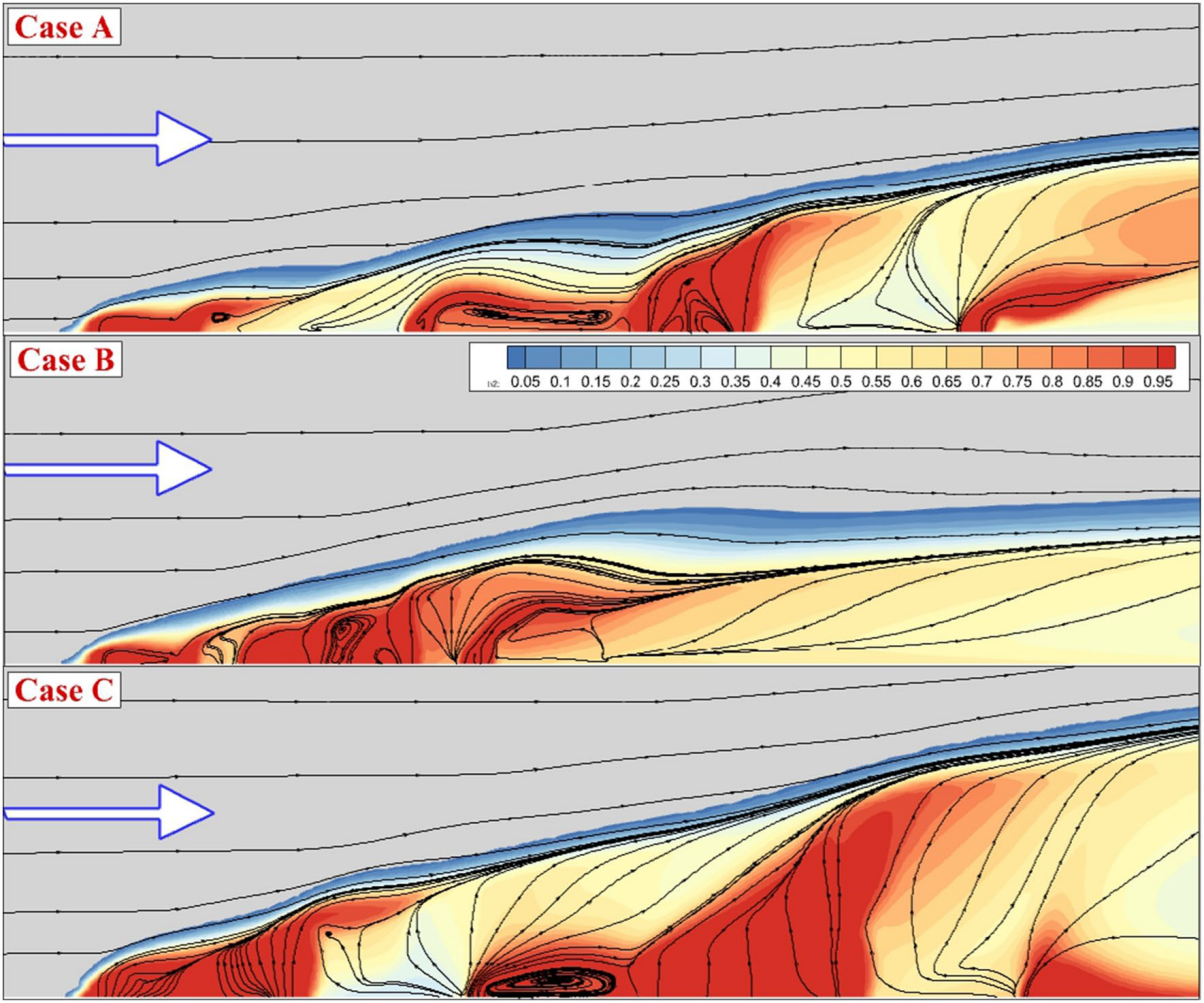
Streamline and fuel concentrations on the jet plane for the annular configurations.

A comparison of the Mach contour on the symmetrical plane for these jet configurations is done in Fig. 4. The two main features related to the supersonic air stream encounter transverse jets are bow shock and shear layer which are specified in Fig. 3. The evaluation of these trails indicates how the nozzle arrangements could efficiently affect on the fuel jet mixing. As the jet space of the proposed model is high (Case A), individual impacts of the jet deflection on the supersonic flow are noticed by produced bow shocks. Meanwhile, the fluctuation of the shear layer also displays the role of the injector configuration on the feature mainstream. In case B, the close gap of the injector unifies the bow shocks, and consequently, the angle of the first bow shock is increased. In case C, which is related to the model with a high aspect ratio of nozzle, the shear layer deflection is less than in case A while the angle of the shear layer grows steadily behind the jets.

**Figure 5:**
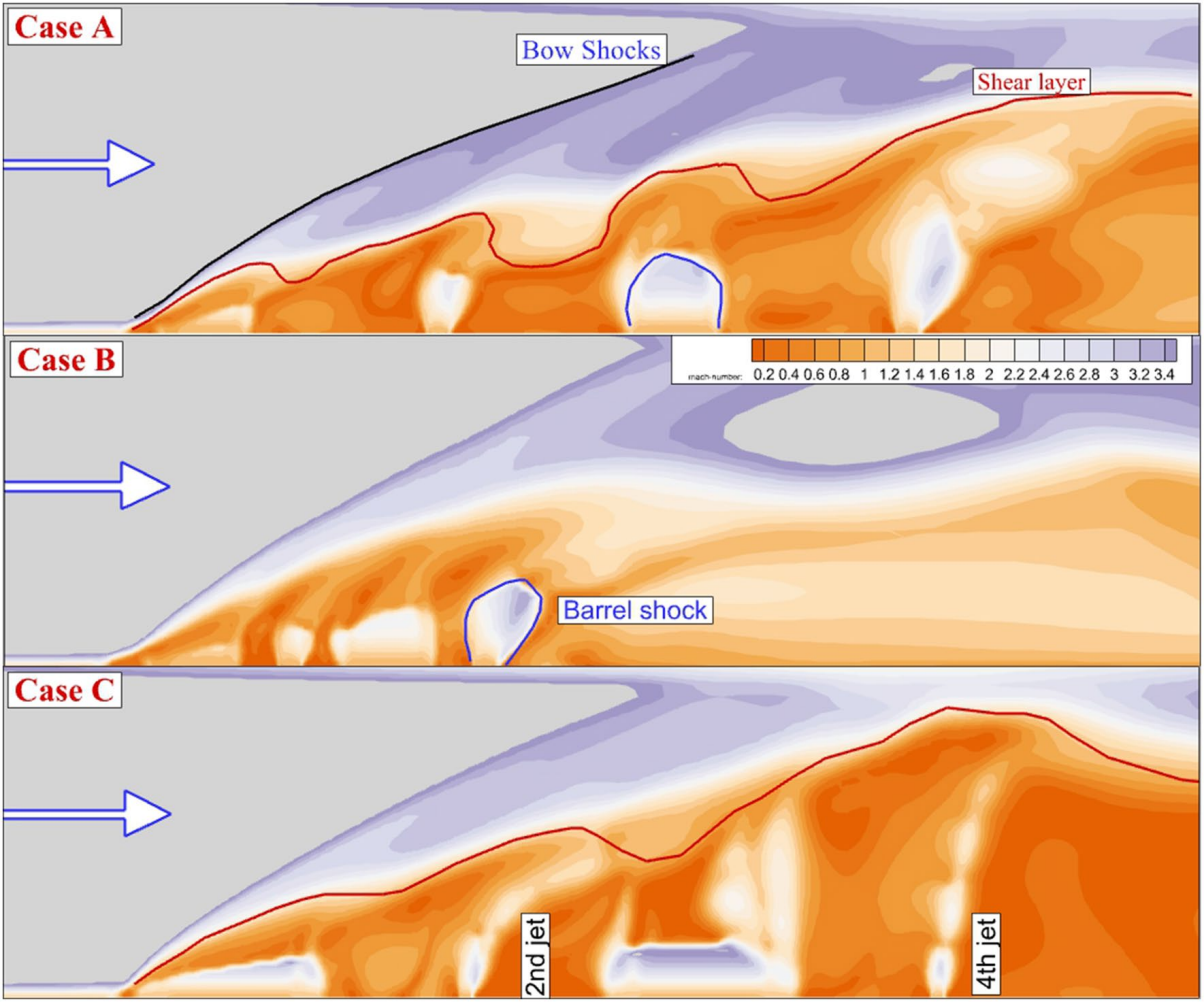
Mach contour on jet plane of annular fuel jet with internal air jet.

Figure 4 illustrates the stream of the jet and main stream as well as fuel jet concentration near the injectors to compare fuel jet interaction on the fuel mixing. In Case A, there is a large gap between the 1st and 2nd injectors as well as the 3rd and 4th injectors. In these gaps, a large circulation with low fuel concentration is produced. To disclose the Impacts of the jet space, Case A and B are compared. Although the concentration of the fuel is higher in Case A, the mixing zone height Is in case B. Comparison of Case A and C indicates the importance of the injector shape on the hydrogen distribution and mixing zone height. As the distribution of the injector along the stream direction is expanded, the height of the mixing zone expands rapidly. Figure 5 displays the Mach contour on the jet planes when the internal air jet is also coupled with the outer fuel jet. The produced barrel.

The produced barrel is extended since the combination of the internal and outer jet produces more momentum near the injector. Indeed, the interaction of the fuel, which is now mixed with inner airflow, intensifies in the new configuration. The difference between the second and fourth injectors with the first and third jet flow becomes clear. One of the important features related to these injector arrangements is the fluctuation of the shear layer which is shown via the red line in Fig. 5. The maximum growth in the shear layer height happens in Case C in which fuel mixing is released in more distance along the combustion chamber. The shape of the barrel shock is also illustrated in the figure.

**Figure 6:**
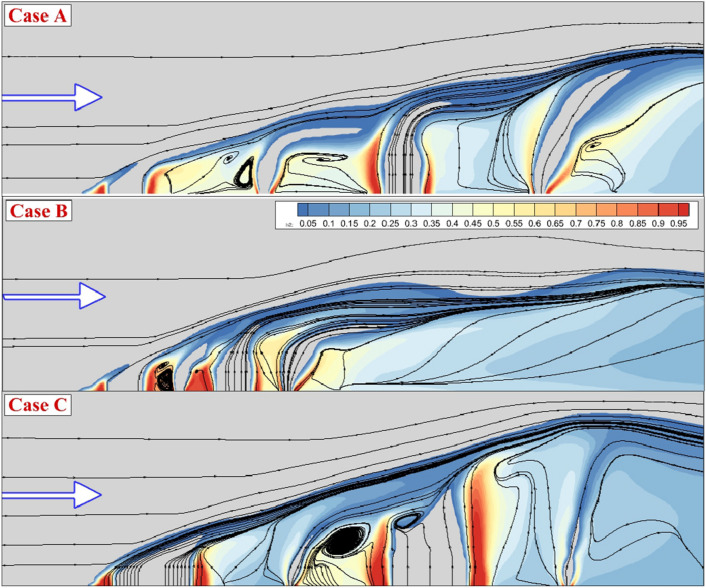
Streamline and fuel concentrations on the jet plane for the annular configurations with internal air jet.

**Figure 7:**
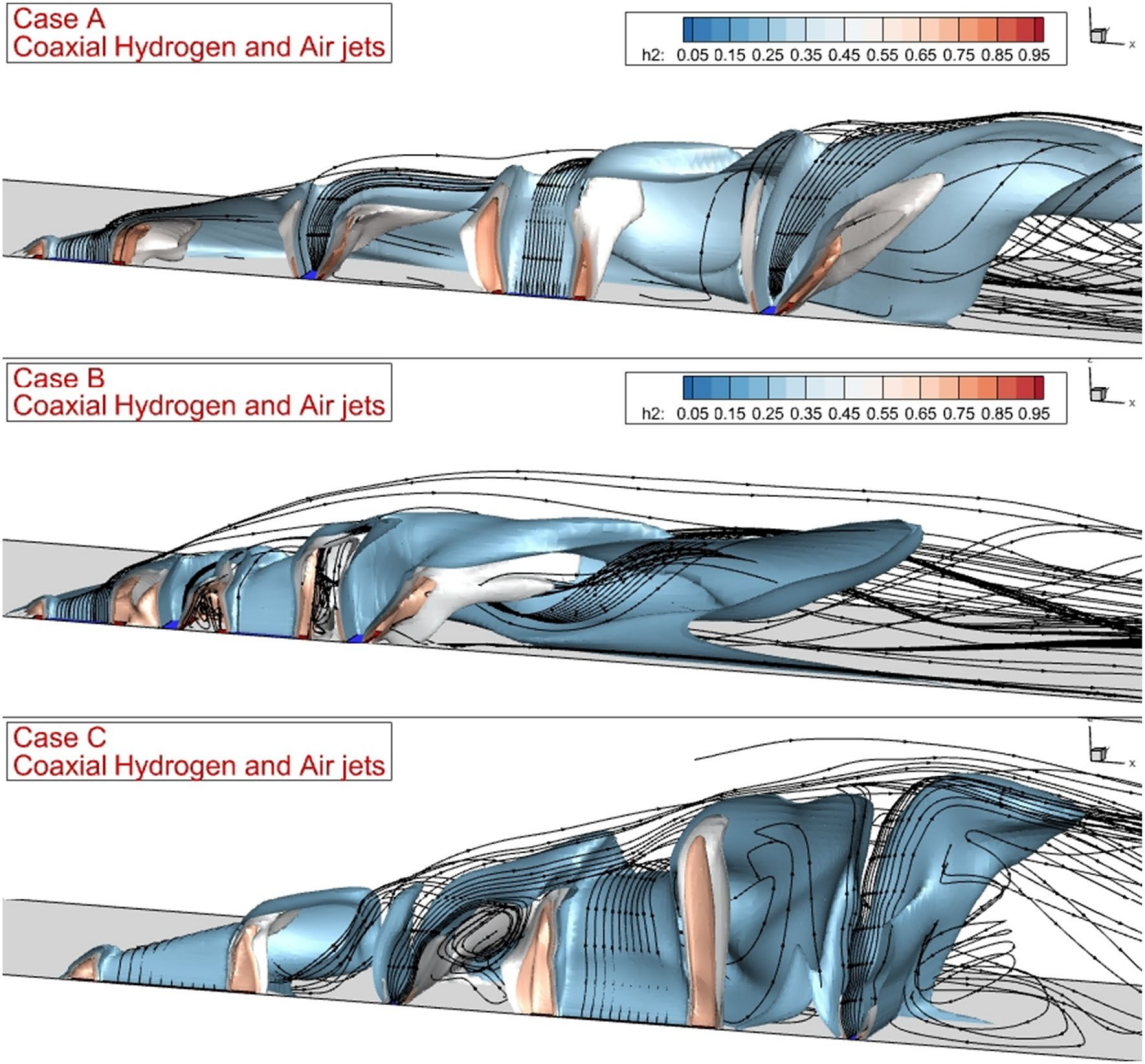
Three-dimensional visualization of the flow for coaxial configurations.

Figure 6 depicts the mixing zone and streamline of the flow when an internal air jet is applied. The higher jet interactions are also expected for this jet configuration and the mixing zone height is more evident in this configuration. A comparison of these models also indicates how jet arrangements could result in higher fuel mixing with more uniform fuel distribution. The three-dimensional flow feature of these jet arrangements is demonstrated in Fig. 7. The jet layers and the contraction of the fuel jet with high-speed airflow are noticed in this image. In case A, the deflection of the air stream is initiated from the first injector as the air jet is coupled with fuel jet. While the deflection of the barrel shock in the first jet is high, the other injectors have less deflection due to the lower interactions. Meanwhile, the formation of the circulation in the gap of the injector is more evident when the space of the jet is reduced in case B. The extension of the injector along the combustor would enhance the formation and production of the circulation in the gap of the nozzles. As the jet is released from the injector with different arrangements, the fluctuations have improved and consequently, the fuel jet could efficiently penetrate the mainstream via this unstable flow structure.

**Figure 8:**
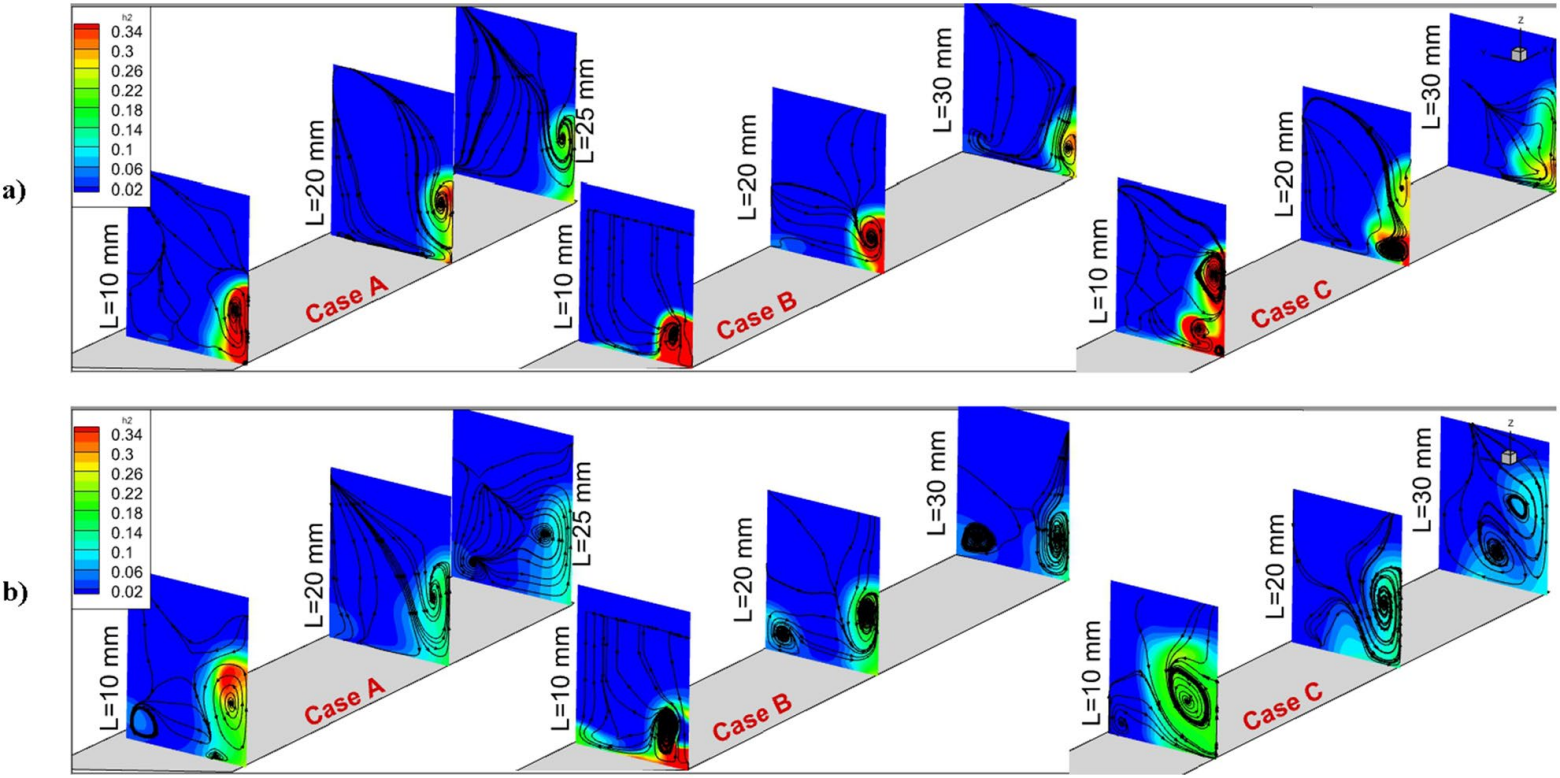
Flow and vortex on the planes behind the injectors (**a**) annular (**b**) coaxial.

Figure 8 compares the flow and circulations behind the injectors to visualize the extension and growth of the hydrogen released from different nozzle arrangements. The significant factor related to these configurations are vortex pair which is initiated from the core of the first injector. The three selected planes are located 10 mm, 20 mm, and 30 mm behind the jets. The core of the injector extends via this vortex pair and the concentrations have decreased since the fuel could effectively penetrate to the main stream. The figure also verifies the importance of the air jet which is released from the core of the jet for the enhancements of the fuel mixing. The production of the secondary vortex is more evident in the models with internal air jets.

**Figure 9:**
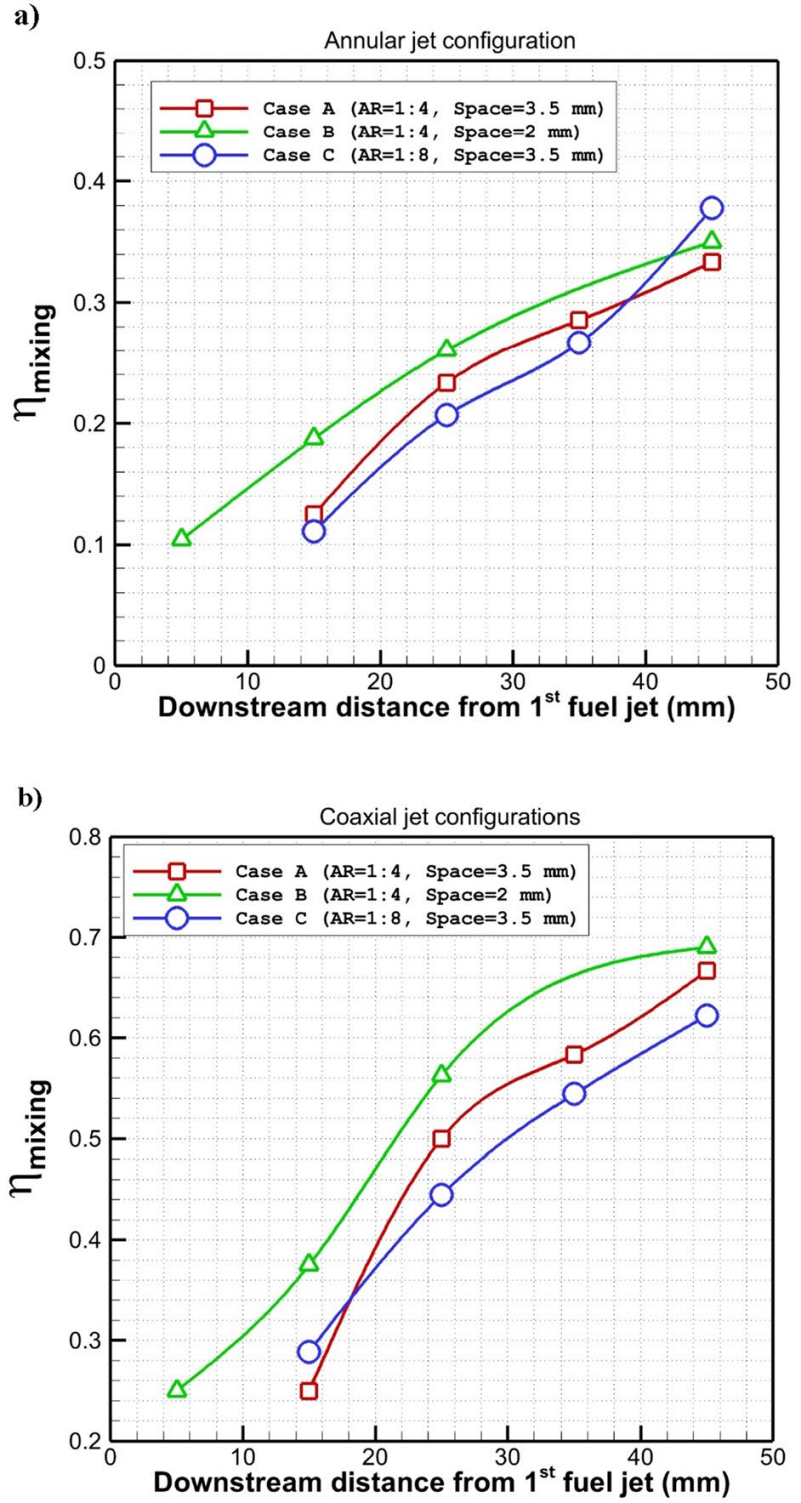
Fuel mixing efficiency behind the jets (**a**) annular (**b**) coaxial.

In Fig. 9a, the changes in the mixing efficiency behind the nozzles in three suggested annular nozzle arrangements are compared. The evaluation of the mixing change along the combustor for the annular injection of these three injectors indicates that reducing the gap of the injector significantly improves the local fuel mixing while the total fuel mixing in far distance is almost identical for these cases. In addition, the increasing of the aspect ratio of rectangular nozzle decreases the local fuel mixing of annular nozzle system while fuel mixing is more efficient in far-distance. The fuel mixing of the coaxial injection system (fuel jet + internal air jet) for the suggested injection system has been displayed in Fig. 9b. The obtained plots indicate that the mixing of the fuel is more efficient when the injector space is lower (case B). Besides, increasing the span of injection (case C) decreases the fuel mixing. Indeed, the interaction of the injector jet decreases in this configuration and consequently, the circulation and fuel penetration are reduced in this model.

**Figure 10:**
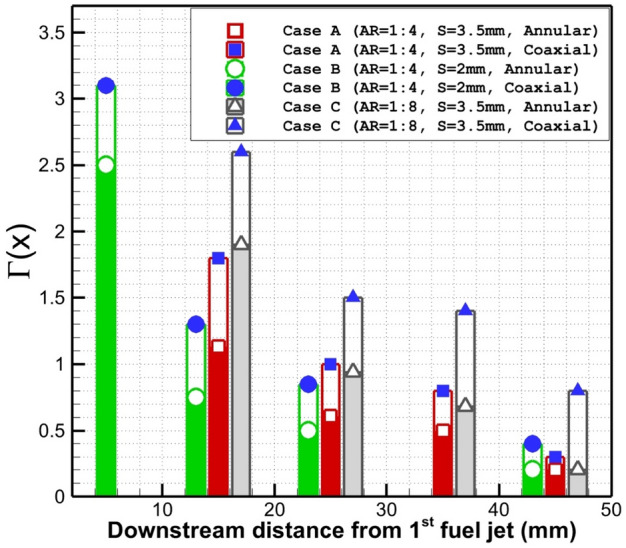
Comparison of the circulation strength of annular and coaxial jet systems.

Figure 10 displays the difference in circulation strength of the annular nozzle with/without the internal air jet. The overall trend of the achieved data indicates that the internal air jet results in higher circulation strength inside the combustor by the reason of the higher jet interactions. A comparison of these models also disclosed that the role of inner air flow is higher when the injector has higher span distribution (case C). The efficiency of the air jet for circulation power is reduced in far distance as demonstrated in Fig. 10.

## Conclusion

In this research article, a numerical study is done to evaluate the fuel jet distribution of the four rectangular injectors in different arrangements at supersonic airflow. This study proposed three arrangements of rectangular injectors for efficient fuel mixing at the transverse supersonic stream. The flow parameters of this injector are fully examined to reveal the most optimum system for the injection of hydrogen inside the combustor. A three-dimensional model of the injectors inside the combustion chamber is used to evaluate the mechanism of the fuel jet released from the annular nozzle in the combustion chamber. The addition of an internal air jet for the enhancement of the fuel jet mixing is also examined in this research study. Obtained results disclosed that the jet arrangement with higher interactions of supersonic airflow and the cross jet results in higher fuel mixing performance in the combustion chamber. The trail of the induced vortex in the combustion chamber for the recommended injector arrangements is also evaluated in this article. Based on the results of the simulations, the trail of vortex pair induced by the core of the jets has a direct impact on the diffusion of hydrogen in the combustor. Fuel mixing is increased in the combustion chamber about 25% by reducing the injector space in this arrangement.

## Data Availability

All data generated or analysed during this study are included in this published article.
